# Enhancing neurorehabilitation by targeting beneficial plasticity

**DOI:** 10.3389/fresc.2023.1198679

**Published:** 2023-06-29

**Authors:** Jonathan R. Wolpaw, Aiko K. Thompson

**Affiliations:** ^1^ National Center for Adaptive Neurotechnologies, Albany Stratton VA Medical Center, Albany, NY, United States; ^2^ Department of Biomedical Sciences, School of Public Health, State University of New York, Albany, NY, United States; ^3^ Department of Health Sciences and Research, College of Health Professions, Medical University of South Carolina, Charleston, SC, United States

**Keywords:** neurorehabilitation, spinal cord injury, targeted plasticity, skilled behavior, heksor, H-reflex conditioning, paired-pulse facilitation, sensorimotor rhythms

## Abstract

Neurorehabilitation is now one of the most exciting areas in neuroscience. Recognition that the central nervous system (CNS) remains plastic through life, new understanding of skilled behaviors (skills), and novel methods for engaging and guiding beneficial plasticity combine to provide unprecedented opportunities for restoring skills impaired by CNS injury or disease. The substrate of a skill is a distributed network of neurons and synapses that changes continually through life to ensure that skill performance remains satisfactory as new skills are acquired, and as growth, aging, and other life events occur. This substrate can extend from cortex to spinal cord. It has recently been given the name “heksor.” In this new context, the primary goal of rehabilitation is to enable damaged heksors to repair themselves so that their skills are once again performed well. Skill-specific practice, the mainstay of standard therapy, often fails to optimally engage the many sites and kinds of plasticity available in the damaged CNS. New noninvasive technology-based interventions can target beneficial plasticity to critical sites in damaged heksors; these interventions may thereby enable much wider beneficial plasticity that enhances skill recovery. Targeted-plasticity interventions include operant conditioning of a spinal reflex or corticospinal motor evoked potential (MEP), paired-pulse facilitation of corticospinal connections, and brain-computer interface (BCI)-based training of electroencephalographic (EEG) sensorimotor rhythms. Initial studies in people with spinal cord injury, stroke, or multiple sclerosis show that these interventions can enhance skill recovery beyond that achieved by skill-specific practice alone. After treatment ends, the repaired heksors maintain the benefits.

## Introduction

The research of the past 50 years has shown that the central nervous system (CNS) changes through life. This plasticity is largely activity-dependent; it is driven by interactions between the CNS and its internal and external environments. It is ubiquitous, has many mechanisms, and occurs everywhere from cortex to spinal cord. Neurons change and new neurons may appear, dendrites and axons change, synapses change, glia change, hormonal effects change, even vasculature changes [see Wolpaw & Kamesar (1) for review and citation of the extensive supporting literature]. The recognition of this lifelong CNS plasticity has made neurorehabilitation one of the most active and exciting areas of biomedical research and clinical practice. Therapeutic results thought impossible a few decades ago are now realistic. As described below, new understanding of how skilled behaviors (skills) such as locomotion, reach-and-grasp, and speech are mastered and then maintained through life leads to new therapeutic strategies for maximizing functional recovery. Furthermore, the therapeutic interventions based on these strategies can take advantage of new biological, bioengineering, and behavioral technologies.

This minireview focuses on one of these new strategies—targeted plasticity. First, it describes how the new understanding of skilled behaviors can account for the frequent failure of standard rehabilitation—primarily task-specific practice—to take full advantage of CNS plasticity. Second, it explains how this new understanding generates the targeted-plasticity strategy. Third, it illustrates the ability of this new strategy to enhance functional recovery in animals and people with spinal cord injury, stroke, or other disorders.

## CNS substrates of skilled behaviors

Extensive research by many groups has clarified three major aspects of skill substrates [see Wolpaw and Kamesar (1) for review and citation of the extensive supporting literature]. First, the substrate of a skill is not stored in a single location, such as a specific cortical area. Rather, it comprises a network of neurons and synapses that may extend from cortex to spinal cord [e.g., (2, 3)]. Second, the neurons and synapses of this network change as needed to preserve skill performance despite the CNS plasticity caused by the acquisition of new skills, and by growth, aging, and other life events. The network's changes are guided by sensory feedback during skill performance and by its outcome (4–7). This process, which may modify the muscle activations and kinematic details of a skill, maintains good performance [e.g., (8)]. Third, because these networks extend from cortex to spinal cord, the networks underlying different skills overlap; they share neurons and synapses. For example, soleus motoneurons and their associated spinal interneurons are not entirely dedicated to locomotion, they participate in many other skills that involve leg muscles. Thus, each network is continually changing in order to maintain its skill despite the changes in the networks underlying other skills. The overall process is a negotiation among them. In the healthy CNS, the networks keep CNS neuronal and synaptic properties in a negotiated equilibrium that enables all of them to produce their skills satisfactorily (1, 9).

These networks that overlap, change continually to maintain their skills, and interact with each other to keep the CNS in a state of negotiated equilibrium, are now called *heksors*. The name *heksor* is derived from an appropriate Greek root (1).

## Common clinical practice in neurorehabilitation: skill-specific practice

Chronic CNS injury or disease, such as spinal cord injury, stroke, or multiple sclerosis, damages the heksors that support important skills and disrupts the negotiated equilibrium of CNS properties that the heksors have established. Thus, the primary goal of rehabilitation is to enable damaged heksors to repair themselves and reestablish a satisfactory negotiated equilibrium. The mainstay of standard rehabilitation therapy is skill-specific practice (e.g., locomotion, reach-and-grasp, speech). It is frequently combined with ancillary activities such as muscle strengthening, range of motion exercises, and coordinated guided movements. While such standard therapy is often effective, many people are left with substantial disabilities [e.g., (10, 11)].

One likely reason for the frequent failure of skill-specific practice to fully restore performance is that the heksor of a skill such as overground walking is very complex (12). It extends from cortex to spinal cord and includes many sites (i.e., neurons and synapses) capable of activity-dependent plasticity. When the locomotion heksor is damaged by a stroke, a spinal cord injury, or other disorder, skill-specific practice might induce plasticity at multiple sites. However, the optimal pattern of plasticity may not occur; and sites capable of beneficial plasticity may not change. For example, rats with unilateral partial SCI regain mobility, but they have an asymmetric gait due to weak stance on the injured side (13, 14). When these rats are exposed to soleus H-reflex up-conditioning on the injured side, H-reflex size increases, the soleus locomotor burst increases, and step symmetry is restored (13, 14). The key takeaway here is that the soleus H-reflex increase, which probably accounts for the increased soleus locomotor burst on the injured side, does not occur *unless* the rat is exposed to H-reflex up-conditioning (13, 14). Simply practicing a defective skill over and over does not guarantee optimal changes in a damaged heksor. Furthermore, such practice may solidify defective performance by the damaged heksor [ (15) for discussion of this issue]. For example, a stroke survivor who practices walking with hip circumduction may simply learn to walk with more robust and consistent hip circumduction.

As the studies reviewed below illustrate, new noninvasive interventions that target beneficial plasticity to critical sites in damaged heksors can induce beneficial plasticity that would not occur without the targeted intervention. Furthermore, this targeted plasticity can improve skill-specific practice, which can lead to wider beneficial plasticity in the damaged heksor and further improve skill performance. In short, the targeted plasticity helps damaged heksors to repair themselves. The result can be more complete skill recovery. This outcome is illustrated in the clinical studies discussed below. In people left with chronic disabilities after standard rehabilitation, the new targeted interventions can produce further clinically significant recovery of function.

## Targeting beneficial plasticity to enhance neurorehabilitation

In the same years that brought new understanding of skilled behaviors, technical advances produced hardware and software for noninvasive interactions with the human CNS that target beneficial plasticity to crucial sites in damaged heksors. This targeted plasticity strategy improves skill-specific practice and leads to wider beneficial plasticity that enhances skill recovery.

The efficacy of targeted plasticity was initially demonstrated in rats with unilateral incomplete spinal cord injuries that had weakened stance on one side, causing asymmetrical locomotion (13). As described above, an operant conditioning protocol that increased the size of the soleus H-reflex on that side strengthened the soleus burst during stance and restored symmetrical locomotion; this improvement did not occur in rats in which the soleus H-reflex was simply elicited without conditioning. A variety of targeted-plasticity interventions are now under study in animals and humans. Here we review the initial clinical results for three of these new interventions. The first targets spinal reflex plasticity; the second corticospinal plasticity; and the third cortical plasticity.

### Operant down-conditioning of the soleus H-reflex

In a controlled study of 13 people with impaired locomotion due to chronic incomplete spinal cord injury (SCI) [American Spinal Injury Association Impairment Scale (AIS) C or D (16)], Thompson et al. (17) showed that down-conditioning of the soleus H-reflex in the more spastic leg resulted in faster and more symmetrical walking. Modulation of EMG activity over the step cycle improved in both legs. In addition, participants reported that they were walking faster and farther in their daily lives, and some noted less clonus, easier stepping, and other improvements. Another reflex conditioning study by another group (18) also reported positive results. A subsequent study of H-reflex down-conditioning during walking in 13 people with chronic incomplete SCI (19) showed that conditioning-induced reflex changes persisted for at least six months after H-reflex down-conditioning ended.

**Figure 1:**
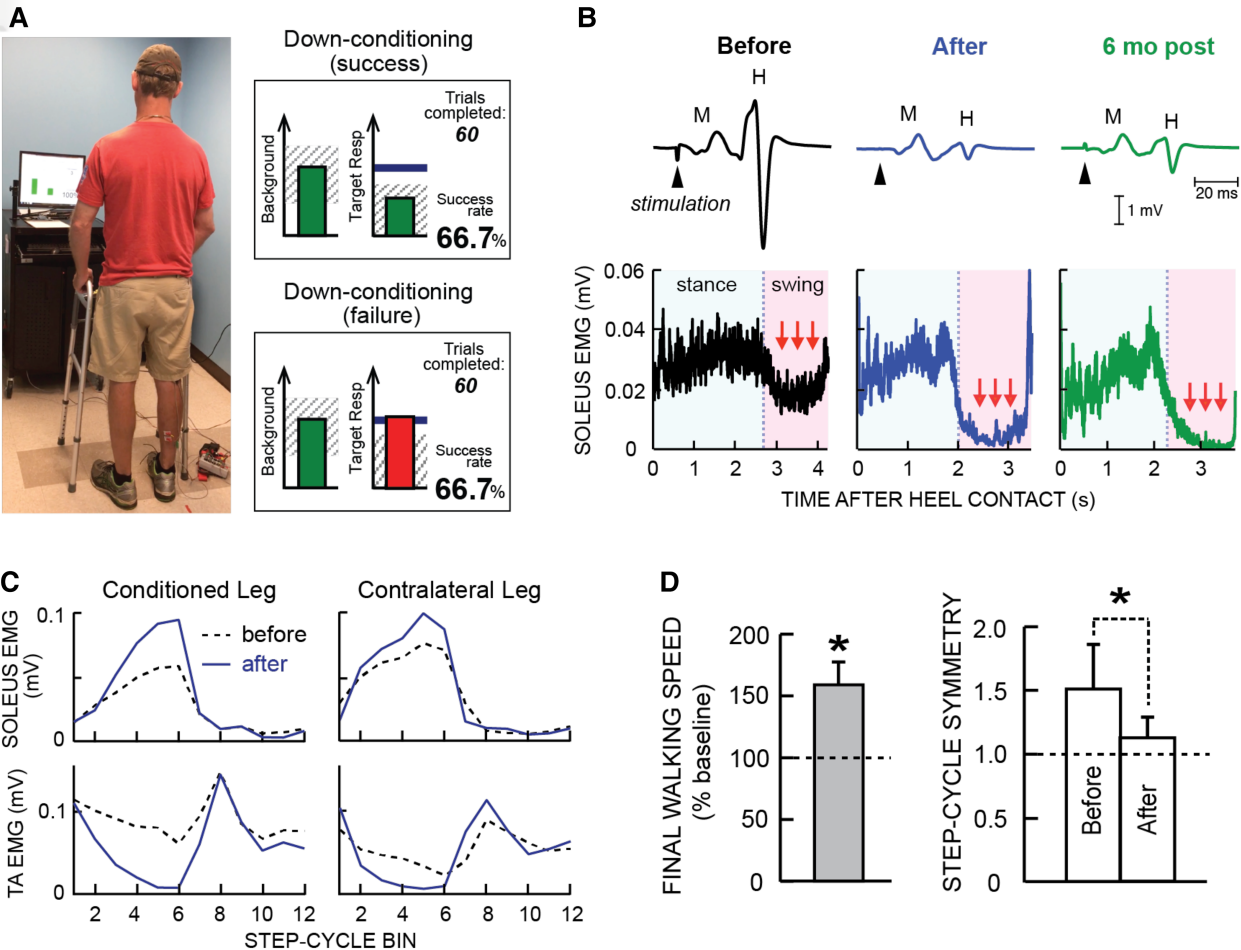
Soleus H-reflex down-conditioning and its effects on walking in people with chronic incomplete spinal cord injury (SCI). (**A**) Reflex conditioning setup and visual feedback. Soleus H-reflexes are elicited while the participant maintains a stable standing posture and stable soleus and tibialis anterior (TA) electromyography (EMG) activity. During reflex trials, the visual feedback screen shows the number of trials completed in the current 75-trial block and ongoing (background) soleus EMG activity (left panel). When soleus EMG stays in the correct (shaded) range for at least 2 s, the tibial nerve is stimulated and an H-reflex is elicited. During control trials (not shown), H-reflex size is not displayed. During conditioning trials, the shading in the Target Response (right) panel indicates the rewarded H-reflex size range for down-conditioning. The dark horizontal line indicates the average H-reflex size for the 6 baseline sessions. The vertical bar (i.e., reflex feedback bar) shows the size of the most recent H-reflex. If H-reflex size falls in the shaded area, the bar is green, and the trial is a success. If it falls outside the shaded area, the bar is red and the trial is a failure. The running success rate for the current 75-trial block is also shown. (**B**) Soleus H-reflex during standing (top) and soleus EMG activity during walking (bottom), measured before and after 30 down-conditioning sessions and then 6 months after conditioning ended in a person with chronic incomplete SCI. Successful conditioning resulted in smaller H-reflexes during standing, and phasic (swing-phase) suppression of soleus EMG activity during walking. The smaller H-reflex and swing-phase suppression of soleus EMG remained present 6 months after conditioning ended. (**C**) Bilateral soleus and TA EMG activity during walking before (dashed line) and after (solid line) 30 down-conditioning sessions in another person with SCI. The step cycle is divided into 12 equal bins, starting from foot contact. Thus, bins 1–7 are the stance phase and bins 8–12 are the swing phase. After successful down-conditioning reduced H-reflex size on the more impaired side, the soleus stance burst was increased and the concurrent inappropriate TA activity was decreased in both legs. Down-conditioning the soleus H-reflex in the more impaired leg improved locomotion in both legs. (**D**) Ten-meter walking speed [mean(±SE) in % of baseline speed] and step-cycle symmetry (mean ± SE) before and after 30 down-conditioning sessions reduced H-reflex size in people with chronic SCI. Step cycle symmetry was measured as the ratio of the time between the nonconditioned leg's foot contact (nFC) and the conditioned leg's foot contact (cFC) to the time between cFC and nFC. Thus, a perfectly symmetrical gait has a value of 1. Initially, the ratio was much greater than 1. After 30 conditioning sessions, the ratio had decreased to just above 1; gait was nearly symmetrical. [(**A**) is modified from Thompson et al. (20)]; (**B–D**) are modified from Thompson et al. (17).

Figure 1 illustrates the soleus H-reflex conditioning protocol and its beneficial effects in people with chronic incomplete SCI. It highlights two crucial aspects of this new therapeutic intervention. First, it produces widespread beneficial plasticity; the locomotor improvements reflect plasticity that extends far beyond the change in the soleus H-reflex pathway. As Figure 1C illustrates, locomotor muscle activity improves in multiple muscles of both legs. A likely explanation for this wider beneficial change is that the targeted change in the H-reflex pathway enabled better walking practice, and this improved practice led to the wider plasticity. Second, the beneficial effects persist after conditioning ends. As Figure 1B illustrates, the smaller H-reflex and improved soleus locomotor EMG were still present at 6-month follow-up. It appears that the restored heksor maintains these beneficial changes. [Furthermore, animal data indicate that the restored heksor may continue to increase these beneficial changes after conditioning ends (21).]

The strategy of inducing targeted plasticity through operant conditioning can be applied to neural pathways other than the soleus H-reflex pathway. For example, operant conditioning of the motor evoked potential (MEP) elicited by transcranial magnetic stimulation (TMS) can target beneficial plasticity to a corticospinal pathway. In people with weak dorsiflexion (foot drop) due to SCI or multiple sclerosis, MEP up-conditioning of the ankle dorsiflexor [i.e., tibialis anterior (TA)] increases TA activity during walking, improves ankle joint motion, and improves walking (22–24).

### Paired corticospinal-motor neuronal stimulation (PCMS)

Perez and her research group have used paired corticospinal-motoneuronal stimulation (PCMS) to induce Hebbian plasticity at corticospinal synapses on motoneurons and thereby strengthen cortical control over the motoneurons (25–29). In a controlled study of 38 people with chronic SCI [American Spinal Injury Association Impairment Scale (AIS) A or B (16)], Jo and Perez (28) tested the effects of 10 sessions of this novel intervention at cervical and lumbar levels in people with chronic spinal cord injuries. Its impact was indicated by the marked increases in motor evoked potentials (MEPs). Furthermore, and most important, the treatment improved both hand-arm function and locomotion, and the improvements persisted six months later.

A subsequent study of 31 people with chronic SCI (29) went on to assess the effects of 20 or 40 PCMS sessions. After 40 sessions, a majority of participants showed improvements in the 10-m walk test that exceeded the minimal clinically important difference (MCID). Furthermore, this functional improvement lasted for at least 9 months after treatment ended.

**Figure 2:**
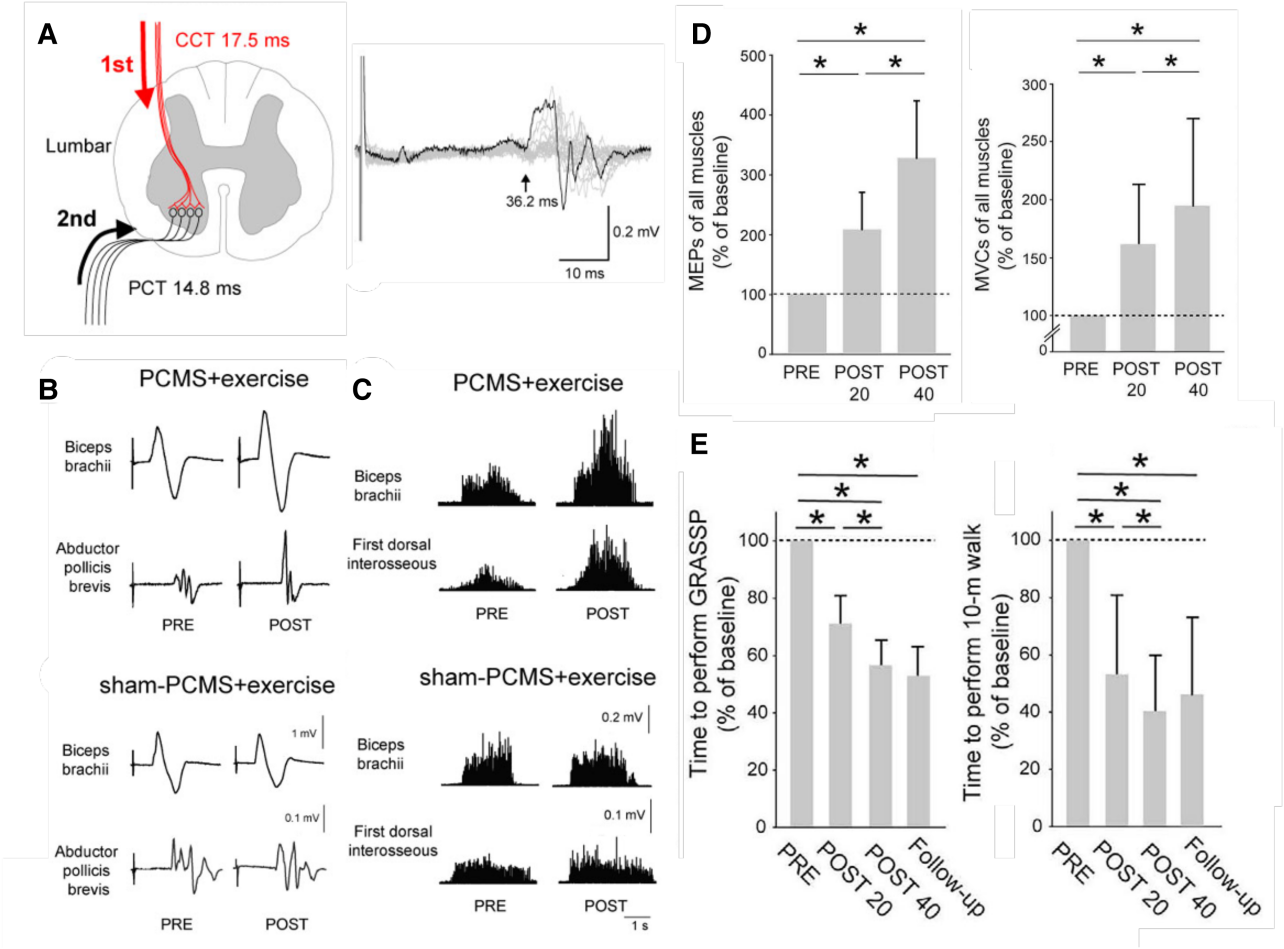
Paired corticospinal-motoneuronal stimulation (PCMS) and its functional effects in people with chronic incomplete spinal cord injury (SCI). (**A**) Left: Central (CCT) and peripheral (PCT) conduction times for tibialis anterior muscle. Stimuli were timed for arrival at corticospinal-motoneuronal synapses by calculating CCT and PPT using latencies from motor evoked potentials (MEPs), F-waves, and M-waves. Right: Tibialis anterior MEP. The arrow indicates latency. (**B**) Average MEPs (30 trials) from biceps brachii and abductor pollicis brevis muscles of representative participants before (pre) and after (post) 10 sessions of exercise plus either PCMS or Sham PCMS. MEPs increased with PCMS, but not with Sham PCMS. (**C**) Maximum voluntary contractions (MVCs) (rectified EMG activity) from biceps brachii and first dorsal interosseous muscles of representative participants before (pre) and after (post) 10 sessions of exercise plus either PCMS or Sham PCMS. MVCs increased with PCMS, but not with Sham PCMS. (**D**) Average (±SD) MEPs (left) and MVCs (right) of all muscles tested for participants before (PRE) and after 20 (POST 20) or 40 (POST 40) sessions of Hebbian stimulation combined with exercise. MEPs and MVCs are significantly increased after 20 sessions and significantly more increased after 40 sessions. (*: *p* < 0.05) [From (29).] **E**: Average (±SD) times to perform the Graded and Redefined Assessment of Strength, Sensibility and Prehension (GRASSP) (left), and the 10-m walk test (right) before and after 20 or 40 sessions and at a 9-month follow-up. The times are significantly decreased after 20 sessions and significantly more decreased after 40 sessions, and the decreases persist at the follow-up 9 months after treatment ends. (*: *p* < 0.05) [From (29).].

Figure 2 illustrates these benefits. Once again, the results indicate that the beneficial plasticity extends beyond the targeted plasticity, and that functional improvements persist after the intervention ends.

### Enhancing electroencephalographic (EEG) correlates of arm/hand function

In a controlled study, Pichiorri et al. (30) studied 28 people with impaired upper extremity function due to a stroke several months earlier. In this controlled study, they used brain-computer interface(BCI)-based feedback to target beneficial plasticity to the corticothalamic circuits responsible for the desynchronization (i.e., decrease) in electroencephalographic (EEG) sensorimotor rhythms (SMRs) that proceeds and accompanies active movements. Greater SMR desynchronization is associated with faster and more accurate movement (31). Thus, the BCI-based feedback sought to increase SMR desynchronization. Combining this targeted plasticity with skill-specific practice produced functional recovery superior to that achieved by skill-specific practice alone.

Norman et al. (32) reported similarly encouraging initial results in people with stroke.

**Figure 3:**
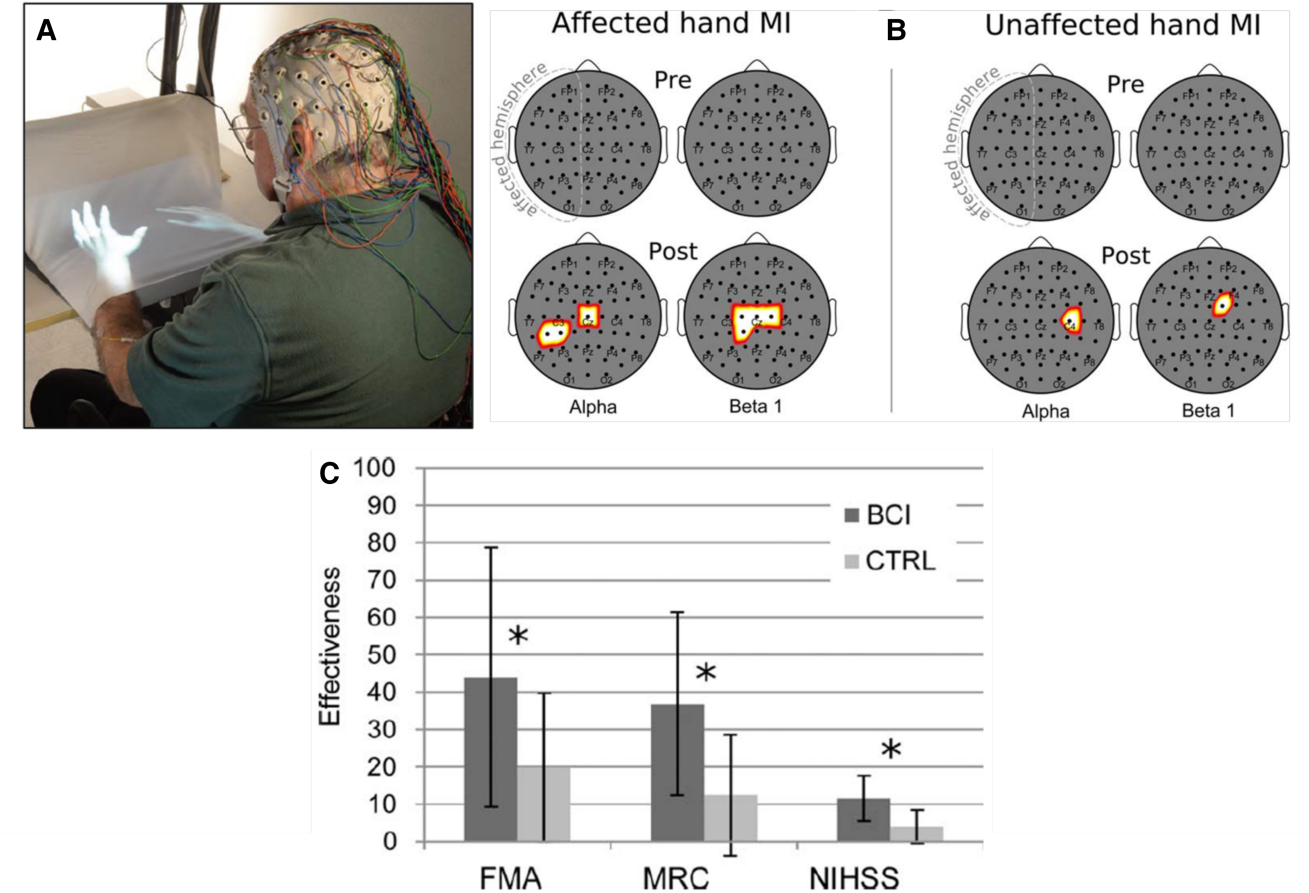
Brain-computer interface (BCI)-based feedback training of motor imagery, and its effects on hand/arm function in people with chronic stroke. (**A**) The patient sits with hands resting on a desk and covered by a white blanket. An adjustable forearm orthosis provides support. The cue and feedback for the patient are projected on the blanket by a custom software program, providing a visual representation of the patient's hands (“virtual hands”). During each session, the therapist monitors the patient's electroencephalogram (EEG) activity continuously through immediate BCI-based feedback displayed on a screen. The amount of sensorimotor rhythm [sensorimotor rhythm (SMR) desynchronization (i.e., decrease)] at selected electrodes determines the vertical velocity of a cursor on the therapist's screen. When the cursor reaches a target in the upper part of the screen, the virtual hand performs the imagined movement (i.e., this is the feedback to the patient for a successful trial). The therapist also monitors electromyography (EMG) activity from the patient's hand and forearm muscles to assess degree of relaxation. (**B**) Statistical scalp maps (nose at top; stroke-affected hemisphere on the left) associated with tonic grasping movement imagery (MI) of the affected (left) and unaffected hands (right). The differences in SMR desynchronization (alpha and beta1 frequency ranges) between the brain–computer interface (BCI) and control (CTRL) patient groups in the Pre (top row) and POST (bottom row) sessions were assessed by *t-*test. Pixel color represents the corresponding probability value. Gray indicates nonsignificant differences; white–yellow indicates stronger desynchronization (*p* < 0.05, Bonferroni corrected) in the BCI group; and red denotes stronger desynchronization (*p* < 0.05, Bonferroni corrected) in the CTRL group. After treatment, SMR desynchronization is greater in the BCI group than in the Control group over the affected hemisphere and, to a lesser extent, over the other hemisphere. (**C**) Improvements in clinical outcome measures [Fugl–Meyer Assessment (FMA), Medical Research Council scale for muscle strength (MRC), National Institute of Health Stroke Scale (NIHSS)] in the BCI group and the Control (CTRL) group. *: *p* < 0.05 between groups by independent-samples *t*-test. The probability of achieving a minimal clinically important difference (MCID) for the FMA (7 points) was significantly (*p* = 0.01) greater in the BCI group (11/14) than in the Control group (3/14). [From (30).].

Figure 3 illustrates the enhanced SMR desynchronization achieved by this BCI-based intervention, and summarizes the substantial associated improvement in hand-arm function as assessed by standard clinical measures. The probability of achieving an MCID in the Fugl-Meyer assessment (7 points) was significantly greater in the BCI group. Once again, targeting beneficial plasticity to a critical part of a damaged heksor led to overall improvement in function.

### Targeted-plasticity interventions in clinical practice

As these first clinical studies show, noninvasive technology-based interventions that target beneficial plasticity to critical sites in damaged heksors are a promising new strategy for enhancing recovery of function. This strategy may be most effective when administered in combination with the mainstay of standard neurorehabilitation, skill-specific practice. The two may be implemented simultaneously [e.g., (30)] or sequentially [e.g., (28)]. The combination is designed to encourage the most important result of the targeted-plasticity strategy—the initiation and enabling of wider beneficial plasticity. The targeted plasticity enables better skill execution and therefore better practice, which may produce beneficial plasticity at many other sites in a damaged heksor.

For example, in Thompson et al. (17) simply reducing the hyperactive soleus reflex led to widespread beneficial plasticity that improved the locomotor participation of multiple muscles in both legs (e.g., Figure 1C). The striking overall improvement in locomotion was not due simply to the smaller soleus H-reflex in one leg; it was due in large part to the much wider plasticity that the H-reflex decrease enabled. By changing the excitability of an important reflex pathway and thereby changing how that pathway functions in general, the targeted plasticity enabled better walking in daily life (e.g., by reducing clonus or footdrop, or restoring right/left step symmetry). This improved practice then drove wider beneficial plasticity.

This wider plasticity is consistent with the finding that the functional improvements produced by combining targeted plasticity with skill-specific practice persist for at least six months after this combined therapy ends; they do not disappear (18, 28, 29). Animal studies provide some insight into this persistence (21). They indicate that, after therapy ends, the damaged heksors maintain, and may even increase the widespread beneficial plasticity. This further supports the concept of a heksor as an active agent that is continually modifying itself to optimize performance of its skill (1). Once guided by the targeting protocol to use the available plasticity more effectively, a damaged heksor may continue to do so after the targeted-plasticity intervention ends (21).

The targeted-plasticity strategy has an essential prerequisite: a clear target. Each of the three studies illustrated in Figures 1–3, satisfied this criterion. In people with spastic hyperreflexia due to incomplete SCI, the excitability of the soleus H-reflex pathway is often abnormally high, which may contribute to an impaired spastic gait (2, 33, 34). Thus, soleus H-reflex down-conditioning was the logical intervention. In Figure 2, strengthening the corticospinal control impaired by incomplete SCI was a similarly logical treatment. And in Figure 3, the established positive correlation between movement-related SMR desynchronization and performance speed and accuracy (31) indicated that increasing the EEG correlate of SMR decrease was the logical intervention.

In some patients, an appropriate target for plasticity may not be obvious, and the therapist may be concerned that an inappropriate target might possibly have harmful effects. Animal studies provide some reassurance in this regard. In rats with weak stance on one side due to incomplete SCI, soleus H-reflex up-conditioning strengthens the stance burst and restores step symmetry (13). However, H-reflex down-conditioning does not weaken the burst and further impair symmetry; the H-reflex decreases, but the stance burst does not (14). It appears that the damaged locomotor heksor changes so as to prevent further locomotor impairment. This adaptation by the locomotor heksor is likely to require adequate sensory feedback (35), which might be impaired by CNS injury or disease. This issue underscores the importance of continuing investigations of the neural mechanisms of impaired motor control.

In the meantime, when an appropriate target is not obvious, guidance may be provided by standard measurements of function at the sites that might be targeted. For example, for a person with stroke who has very strong flexor synergies and large H-reflexes in wrist flexors, down-conditioning a flexor H-reflex might be effective [just as soleus H-reflex down-conditioning was effective in people with spasticity and impaired gait due to SCI (17, 18)]. In contrast, for a person with stroke who has an abnormally small MEP in wrist extensors, up-conditioning the extensor MEP might be considered appropriate (22–24).

With continued development, targeted-plasticity protocols should be applicable to a variety of sites important in sensorimotor function. For example, reciprocal inhibition has been successfully conditioned in rats (36). Other spinal reflexes, other ascending or descending spinal pathways, and other EEG rhythms might be targeted by operant conditioning or other protocols. In the future, a targeted-plasticity intervention might be highly personalized; it might be configured to address a patient's specific deficits.

## Conclusion

Skilled behaviors are produced and maintained by widely distributed networks of neurons and synapses now called heksors. Heksors change continually through life to maintain satisfactory performance of their skills. The primary goal of neurorehabilitation is to enable damaged heksors to repair themselves and regain satisfactory skill performance. Skill-specific practice plus ancillary exercises—the mainstay of standard rehabilitation—does not ensure optimal engagement of the many sites and kinds of plasticity present in the CNS. Thus, heksor plasticity that could improve performance may not occur. Noninvasive technology-based interventions that target beneficial plasticity to critical sites in damaged heksors can improve skill-specific practice and thereby increase the efficacy of standard rehabilitation therapy. The first clinical studies indicate that these new targeted-plasticity interventions can complement standard rehabilitation and enhance recovery of function for people with SCI, stroke, or multiple sclerosis. Most important, the benefits persist after the intervention ends. Just as they adapted as needed to maintain their skills prior to CNS injury, the repaired heksors maintain the beneficial effects initiated by the targeted interventions.
